# Relationship between Plasma Leptin Level and Chronic Kidney Disease

**DOI:** 10.1155/2012/269532

**Published:** 2012-05-14

**Authors:** Anoop Shankar, Shirmila Syamala, Jie Xiao, Paul Muntner

**Affiliations:** ^1^Department of Community Medicine, West Virginia University School of Medicine, P.O. Box 9190, Morgantown, WV 26506-9190, USA; ^2^Department of Medicine, West Virginia University School of Medicine, Morgantown, WV 26506, USA; ^3^Department of Epidemiology, University of Alabama at Birmingham, Birmingham, AL 35294, USA

## Abstract

*Background*. Leptin is an adipose tissue-derived hormone shown to be related to several metabolic, inflammatory, and hemostatic factors related to chronic kidney disease. Recent animal studies have reported that infusion of recombinant leptin into normal rats for 3 weeks fosters the development of glomerulosclerosis. However, few studies have examined the association between leptin and CKD in humans. Therefore, we examined the association between plasma leptin levels and CKD in a representative sample of US adults. *Methods*. We examined the third National Health and Nutrition Examination Survey participants >20 years of age (*n* = 5820, 53.6% women). Plasma leptin levels were categorized into quartiles (≤4.3 Fg/L, 4.4–8.7 Fg/L, 8.8–16.9 Fg/L, >16.9 Fg/L). CKD was defined as a glomerular filtration rate of <60 mL/min/1.73 m^2^ estimated from serum creatinine. *Results*. Higher plasma leptin levels were associated with CKD after adjusting for age, sex, race/ethnicity, education, smoking, alcohol intake, body mass index (BMI), diabetes, hypertension, and serum cholesterol. Compared to quartile 1 of leptin (referent), the odds ratio (95% confidence interval) of CKD associated with quartile 4 was 3.31 (1.41 to 7.78); *P-trend* = 0.0135. Subgroup analyses examining the relation between leptin and CKD by gender, BMI categories, diabetes, and hypertension status also showed a consistent positive association. *Conclusion*. Higher plasma leptin levels are associated with CKD in a representative sample of US adults.

## 1. Introduction

Leptin is an adipose tissue-derived hormone that has been shown to be related to several metabolic, inflammatory, and hemostatic factors involved in the development of hypertension and cardiovascular disease [1]. Experimental animal studies suggest that higher leptin levels may cause hyperglycemia, elevations in blood pressure (mediated through increased sympathetic activity), and renal dysfunction [2]. In rat models, leptin has been shown to induce natriuresis [3] which may in turn result in an increase in arterial pressure so as to maintain sodium and water balance [4]. Leptin has also been shown to serve as a cofactor of TGF-beta activation, promote renal endothelial cell proliferation, and potentially may play a role in renal glomerulosclerosis [5–7]. Recent studies have reported that infusion of recombinant leptin into normal rats for 3 weeks fosters the development of focal glomerulos 4.clerosis [5]. However, few human studies have examined the putative association between plasma leptin levels and chronic kidney disease (CKD) in humans. In this context, we examined the independent relation between plasma leptin levels and CKD in a multiethnic sample of US adults, after adjusting for main confounding factors.

## 2. Methods

The current study is based on data from the Third National Health and Nutrition Examination Survey (NHANES III). Detailed description of NHANES III study design and methods are available elsewhere [8–13]. In brief, NHANES III included a stratified multistage probability sample representative of the civilian noninstitutionalized US population. Selection was based on counties, blocks, households, and individuals within households and included the oversampling of non-Hispanic blacks and Mexican Americans in order to provide stable estimates of these groups. Subjects were required to sign a consent form before their participation, and approval was obtained from the Human Subjects Committee in the US Department of Health and Human Service. Secondary data analysis was approved by the West Virginia University Institutional Review Board.

The sample included in the current analysis consisted of participants aged greater than 20 years who were randomly assigned to complete an examination in the morning after an overnight fast. Plasma leptin levels were measured in 6415 of these participants. We further excluded participants with self-reported cardiovascular disease (*n* = 434), missing serum creatinine (*n* = 60) or who were missing data (*n* = 101) on covariates included in the multivariable model, including systolic or diastolic blood pressure, body mass index (BMI), or cholesterol levels. This resulted in 5820 participants (53.6% women).

### 2.1. Main Outcome of Interest: Presence of Chronic Kidney Disease

 Serum creatinine was measured using the Jaffe kinetic alkaline picrate method performed on a Roche Hitachi 737 analyzer [13]. The laboratory coefficient of variability ranged from 0.2% to 1.4%. Serum creatinine values in NHANES III were calibrated to the standard creatinine values from the Cleveland Clinic Foundation (CCF) laboratory who used a Roche coupled enzymatic assay method that was traceable to an isotope dilution mass spectrometric method using the following Deming regression equation: Standard Creatinine in mg/dL = 0.960 × NHANES measured serum creatinine (in mg/dL)–0.184 [14]. Glomerular filtration rate (eGFR) was estimated from serum creatinine using the 4-variable Modification of Diet in Renal Disease (MDRD) study equation as follows: eGFR =175 × (serum creatinine in mg/dL)^−1.154^ × (age in years)^−0.203^ × (0.742 if female) × (1.21 if black) [15]. CKD was defined as an eGFR of <60 mL/min/1.73 m^2^, consistent with National Kidney Foundation Kidney Disease Outcomes Quality Initiative (KDOQI) ≥  Stage 3 chronic kidney disease [16]. 

### 2.2. Exposure Measurements

 Age, gender, race/ethnicity, smoking status, alcohol intake (g/day), level of education, history of diabetes and oral hypoglycemic intake or insulin administration, and antihypertensive medication use were assessed using standardized questionnaires. Individuals who had not smoked ≥100 cigarettes in their lifetimes were considered never smokers; those who had smoked ≥100 cigarettes in their lifetimes were considered former smokers if they answered negatively to the question “Do you smoke now?” and current smokers if they answered affirmatively. Using height and weight measured during the study examination, body mass index (BMI) was calculated as weight in kilograms divided by height in meters squared.

 Rigorous procedures with quality control checks were used in blood collection and details about these procedures are provided in the NHANES Laboratory/Medical Technologists Procedures Manual [8, 11]. Measurement of plasma leptin was performed by Linco Research, Inc., St. Louis, Mo, USA. The assay was a radioimmunoassay (RIA) with a polyclonal antibody raised in rabbits against highly purified recombinant human leptin. The minimum detectable concentration of the assay was 0.5 Fg/L leptin, and the limit of linearity was 100 Fg/L. Recovery of leptin added to serum is 99–104% over the linear range of the assay. The RIA agrees reasonable well with rough quantification by Western blot. Within- and between-assay CVs ranged from 3.4% to 8.3% and from 3.6% to 6.2%, respectively [11, 12].

 Serum total cholesterol was measured enzymatically at the Johns Hopkins Lipid Laboratory. Serum glucose was measured using the modified hexokinase method at the University of Missouri Diabetes Diagnostic Laboratory. Diabetes was defined based on the guidelines of the American Diabetes Association as a serum glucose ≥126 mg/dL after fasting for a minimum of 8 hours, a serum glucose ≥200 mg/dL for those who fasted <8 hours before their NHANES visit, a glycosylated hemoglobin value ≥6.5%, or a self-reported current use of oral hypoglycemic medication or insulin. Seated systolic and diastolic blood pressures were measured using a mercury sphygmomanometer according to the American Heart Association and Seventh Joint National Committee (JNC7) recommendations [17]. Up to 3 measurements were averaged for systolic and diastolic pressures. Participants were considered to have hypertension if they reported current blood pressure-reducing medication use and/or had systolic blood pressure ≥140 mm Hg and/or diastolic blood pressure ≥90 mm Hg [17].

### 2.3. Statistical Analysis

Plasma leptin was analyzed both as a continuous variable as well as a categorical variable. For the analysis as a continuous variable, leptin values were log transformed (base e) as a result of their skewed distribution. Using the distribution present in the NHANES III population, we categorized plasma leptin level into quartiles (≤4.3 Fg/L, 4.4–8.7 Fg/L, 8.8–16.9 Fg/L, >16.9 Fg/L). The multivariable-adjusted odds ratio [(OR) (95% confidence interval (CI)] of CKD associated with leptin quartile was calculated with the lowest quartile as the referent, using logistic regression models. Odds ratios were calculated initially after age and sex adjustment and subsequently after additional adjusting for race/ethnicity (non-Hispanic whites, non-Hispanic blacks, Mexican Americans, and others), education categories (below high school, high school, above high school), smoking (never smoker, former smoker, current smoker), alcohol intake (continuous), BMI (continuous), diabetes mellitus (absent, present), hypertension (absent, present), and total serum cholesterol (continuous). Trends in the OR of CKD across increasing plasma leptin category were determined by modeling median within-quartile leptin level as a continuous variable. To examine the dose-response relationship of the observed association between plasma leptin level and CKD without linearity assumptions, we used flexible nonparametric logistic regression employing the generalized additive modeling approach (R system for statistical computing, available from Comprehensive R Archive Network [http://www.cran.r-project.org/]) to calculate odds ratio of CKDs mellitus, adjusting for all covariates in the multivariable model; the predicted odds ratio of CKD was then plotted against increasing leptin levels (on the log scale). Previous studies have shown that serum leptin levels are associated with increased systemic inflammation as measured by C-reactive protein levels [18], hyperglycemia [12], high insulin levels [19], and increased systolic blood pressure [20], factors that have also been shown to be associated with CKD[17, 21, 22]. Therefore, in a supplementary analysis, to examine if the observed association between plasma leptin and CKD was explained by C-reactive protein levels, fasting insulin, glucose levels, or systolic blood pressure, we adjusted for these variables in the multivariable-adjusted model. Sample weights [9] that account for the unequal probabilities of selection, oversampling, and nonresponse were applied for all analyses using SUDAAN (version 8.0; Research Triangle Institute, Research Triangle Park, NC) and SAS (version 9.2; SAS Institute, Cary, NC) software; Standard errors (SE) were estimated using the Taylor series linearization method.

## 3. Results

Table 1 presents the characteristics of the study population included in the current analysis. Overall, this study included a broad age range, multiethnic sample of Americans with an approximately equal number of men and women. The mean eGFR of the study participants was 94 mL/min/1.73 m^2^, and 3.5% had CKD.

Table 2 presents the association between quartile of plasma leptin and CKD. A positive association between higher leptin quartiles and CKD was present in the age, sex-adjusted model as well as the multivariable model. When analyzed as a continuous variable after log transformation, a positive association was present between leptin and CKD.

Table 3 presents the association between plasma leptin levels and CKD within subgroups defined by gender, BMI categories, and diabetes and hypertension. Overall, the association between leptin and CKD was consistently present within these subgroups. Although some of the ORs failed to reach conventional levels of statistical significance due to limited sample size and therefore statistical power, tests for interaction were not statistically significant (each *P* > 0.10 for all stratified analyses).

**Figure 1:**
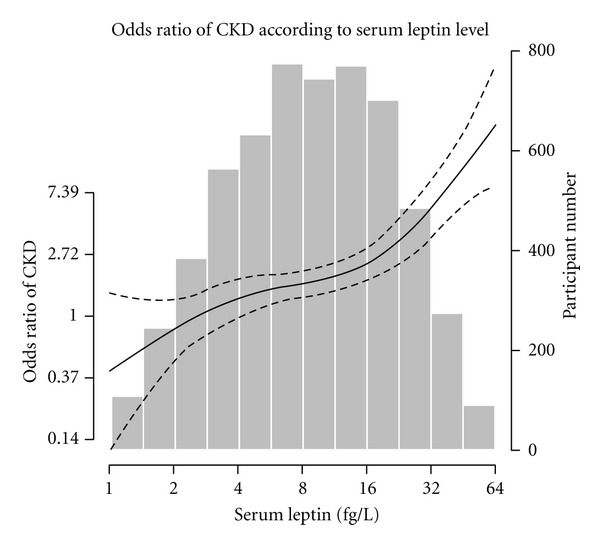
Multivariable-adjusted odds ratio of chronic kidney disease (CKD) according to plasma leptin level (Fg/L). Solid thick line represents the predicted odds of CKD from nonparametric logistic regression; dashed lines, 95% confidence limits for the nonparametric logistic regression estimates. The nonparametric logistic regression was adjusted for age (years), sex (men, women), race-ethnicity (non-Hispanic whites, non-Hispanic blacks, Mexican Americans, others), education categories (<high school, high school, >high school), smoking (never, former, current), body mass index (normal, overweight, obese), diabetes (absent, present), and serum total cholesterol (mg/dL). The median value of quartile 1 of leptin (1.285 Fg/L) was used as the referent category to calculate odds ratios. *X* axis: plasma leptin level (fg/L) plotted in log scale, *Y*1 axis: predicted odds ratio of CKD plotted in log scale, *Y*2 axis: participant number for each serum plasma leptin level.

 When we employed nonparametric models to graphically examine the dose-response relationship between plasma leptin levels and CKD without linearity assumptions involved in traditional regression models, we observed an overall positive association between plasma leptin and CKD, consistent with the results in Tables 1, 2, and 3. However, there was a steeper association with CKD for plasma leptin levels >16 Fg/L (Figure 1).

 In a supplementary analysis, to examine if the observed association between leptin and CKD was explained by C-reactive protein, a marker of inflammation, or fasting insulin or glucose levels, or systolic blood pressure, we additionally adjusted for these variables to the multivariable-adjusted model. The positive association between leptin and CKD was attenuated, but still present. Compared to quartile 1 of plasma leptin (referent), the multivariable OR (95% CI) of CKD was 1.31 (0.70 to 2.44) in quartile 2, 1.22 (0.57 to 2.62) in quartile 3, and 2.72 (1.14 to 6.48) in quartile 4; *P*-trend = 0.0475.

## 4. Discussion

 In a multi-ethnic, population-based sample of US adults, we found that higher plasma leptin levels were positively associated with CKD. This association appeared to be independent of confounders such as age, race-ethnicity, education, BMI, diabetes, and hypertension and appeared to be consistently present in both men and women. Furthermore, the observed association between plasma leptin levels and CKD was present even after adjusting for C-reactive protein and fasting insulin levels, suggesting an association between this adipokine and CKD that is independent of these factors.

 Several lines of recent evidence suggest that an association between leptin and CKD is plausible. This includes the role of leptin in activating the sympathetic nervous system and causing chronic elevations in blood pressure and renal dysfunction [2], inducing natriuresis [3] which may result in an increase in arterial pressure so as to maintain sodium and water balance [4], serving as a cofactor of TGF-beta activation, promoting renal endothelial cell proliferation, and subsequent glomerulosclerosis [5–7]. It was recently shown that, in rats, infusion of recombinant leptin caused the development of focal glomerulosclerosis [5]. Also, leptin is also reported to be related to insulin resistance [23] and high C-reactive protein levels [24], both of which have been shown to be related to CKD [21, 25].

 There are a few studies in the literature for comparison. A previous study conducted among women with type 1 diabetes reported that plasma leptin levels are independently related to reduced renal function [26]. In a study from South Africa conducted among approximately 300 black subjects, Okpechi et al. reported that plasma leptin levels were inversely related to eGFR [27]. In another study, common polymorphisms in the *LEP *gene were found to be associated positively with serum creatinine and inversely with eGFR [28]. Overall, our findings of a positive association between plasma leptin and CKD are in agreement with these previous studies; in addition, we were able to study a large multiethnic sample that includes both men and women and also adjusts for multiple confounders such as BMI, diabetes, hypertension, lipid levels, and C-reactive protein.

 The main strengths of our study include its population-based nature, inclusion of a representative multiethnic sample, adequate sample size, and the availability of data on confounders for multivariable adjustment. Furthermore, all data were collected following rigorous methodology, including a study protocol with standardized quality control checks. The main limitation of our study is the cross-sectional nature of NHANES, which precludes conclusions regarding the temporal nature of the association between plasma leptin and CKD. Second, defining CKD as eGFR <60 mL/min/1.73 m^2^ introduces some ascertainment bias. This bias is likely to result in under- or overestimation of odds ratios presented in this report.

 In summary, in a multiethnic sample of US adults, we found that higher plasma leptin levels are associated with CKD, independent of traditional factors such as age, sex, smoking, alcohol intake, BMI, diabetes, hypertension and serum cholesterol. Our results suggest that leptin may explain part of the reported association between obesity and kidney disease. However, future prospective cohort studies are needed to confirm or refute our findings.

## Figures and Tables

**Table 1:** Characteristics of the study population (*n* = 5820).

Characteristics	Mean or percentage ± standard error
Age, years	43.1 ± 0.5
Women, %	53.6 ± 0.9
Race-ethnicity, %	
Non-Hispanic whites	77.1 ± 1.5
Non-Hispanic blacks	10.3 ± 0.7
Mexican Americans	5.1 ± 0.5
Other	7.6 ± 1.0
Education categories, %	
Less than high school	21.5 ± 1.1
High school	34.3 ± 1.1
More than high school	44.2 ± 1.5
Smoking, %	
Never	48.3 ± 1.0
Former	25.0 ± 0.7
Current	26.7 ± 1.0
Current alcohol drinker, %	56.1 ± 1.7
Body mass index, kg/m^2^	26.5 ± 0.2
Diabetes mellitus, %	5.0 ± 0.4
Hypertension, %	28.4 ± 1.1
Total cholesterol, mg/dL	203.0 ± 0.9
Glomerular filtration rate, mL/min/1.73 m^2^	95.6 ± 0.7
Chronic kidney disease, %*	3.6 ± 0.3
Glucose, mg/dL	98.8 ± 0.4
Insulin, uU/mL	10.4 ± 0.2
Systolic blood pressure, mmHg	120.8 ± 0.5
Diastolic blood pressure, mmHg	73.8 ± 0.2
C-reactive protein, mg/dL	0.38 ± 0.01

*Defined as estimated glomerular filtration rate <60 mL/min/1.73 m^2^.

**Table 2:** Association between plasma leptin level and prevalence of chronic kidney disease (CKD).

Plasma leptin level	*N*	CKD weighted %	Age, sex-adjusted odds ratio (95% confidence interval)	Multivariable-adjusted odds ratio (95% confidence interval)*
Quartile 1 (≤4.3 Fg/L)	1453	1.8	1 (referent)	1 (referent)
Quartile 2 (4.4–8.7 Fg/L)	1541	3.2	1.42 (0.80 to 2.52)	1.35 (0.73 to 2.52)
Quartile 3 (8.8–16.9 Fg/L)	1464	3.5	1.40 (0.74 to 2.62)	1.34 (0.63 to 2.87)
Quartile 4 (>16.9 Fg/L)	1452	6.3	3.25 (1.61 to 6.55)	3.31 (1.41 to 7.78)
*P*-trend			0.0019	0.0135

Log-transformed leptin	5820	3.6	1.57 (1.23 to 2.01)	1.74 (1.27 to 2.38)

*Adjusted for age (years), sex (men, women), race-ethnicity (non-Hispanic whites, non-Hispanic blacks, Mexican Americans, others), education categories (<high school, high school, >high school), smoking (never, former, current), alcohol intake (never, former, current), body mass index (normal, overweight, obese), diabetes (absent, present), hypertension (absent, present), and serum total cholesterol (mg/dL).

**Table 3:** Association between plasma leptin level and chronic kidney disease (CKD), by subgroups.

Subgroups of interest	No. at risk	CKD weighted %	Multivariable-adjusted odds ratio of CKD associated with log-leptin (95% confidence interval)*
Age			
<60	4324	0.9	2.06 (1.01 to 4.20)
≥60	1496	15.7	1.36 (1.01 to 1.83)

Gender			
Men	2627	2.8	1.43 (0.96 to 2.12)
Women	3193	4.3	1.77 (1.09 to 2.88)

Race-ethnicity, %			
Non-Hispanic whites	2403	3.9	1.63 (1.10 to 2.41)
Non-Hispanic blacks	1615	2.5	3.31 (1.86 to 5.90)
Mexican Americans, others	1802	2.5	1.40 (0.80 to 2.45)

Body mass index (BMI)			
BMI <25 kg/m^2^	2273	2.9	1.38 (0.62 to 3.07)
25 kg/m^2^≤ BMI <30 kg/m^2^	2058	3.6	2.44 (1.48 to 4.04)
BMI ≥30 kg/m^2^	1489	4.9	3.30 (1.91 to 5.70)

Diabetes			
Absent	5358	3.2	1.70 (1.23 to 2.35)
Present	462	10.1	1.41 (0.42 to 4.73)

Hypertension			
Absent	3908	1.6	1.42 (0.66 to 3.07)
Present	1912	8.6	1.73 (1.20 to 2.49)

*Adjusted for age (years), sex (female, male), race-ethnicity (non-Hispanic whites, non-Hispanic blacks, Mexican Americans, others), education categories (<high school, high school, >high school), smoking (never, former, current), current alcohol intake (absent, present), body mass index (normal, overweight, obese), diabetes (absent, present), hypertension (absent, present), serum total cholesterol (mg/dL), glucose (mg/dL), insulin (uU/mL) and C-reactive protein (mg/dL), except the stratifying variable.
